# The Hinge Region of Bovine Zona Pellucida Glycoprotein ZP3 Is Involved in the Formation of the Sperm-Binding Active ZP3/ZP4 Complex

**DOI:** 10.3390/biom5043339

**Published:** 2015-11-23

**Authors:** Kaori Suzuki, Nanami Tatebe, Sayuri Kojima, Ayumi Hamano, Misaki Orita, Naoto Yonezawa

**Affiliations:** Graduate School of Science, Chiba University, Chiba 263-8522, Japan; E-Mails: k.suzuki.0116@gmail.com (K.S.); ttb7mm@gmail.com (N.T.); kojikojiko1115@yahoo.co.jp (S.K.); hama_0315@graduate.chiba-u.jp (A.H.); aana2013@chiba-u.jp (M.O.)

**Keywords:** zona pellucida, fertilization, glycoprotein, baculovirus-Sf9 cell

## Abstract

The zona pellucida (ZP) surrounds the mammalian oocyte and mediates species-selective sperm-oocyte interactions. Bovine ZP consists of glycoproteins ZP2, ZP3, and ZP4. Neither ZP3 nor ZP4 alone shows inhibitory activity for the binding of sperm to the ZP; however, this activity is seen with the ZP3/ZP4 heterocomplex. Here, we constructed a series of bovine ZP3 mutants to identify the ZP4-binding site on ZP3. Each ZP3 mutant was co-expressed with ZP4 using a baculovirus-Sf9 cell expression system and examined for interaction with ZP4 as well as inhibitory activity for sperm-ZP binding. *N*-terminal fragment Arg-32 to Arg-160 of ZP3 interacted with ZP4 and inhibited sperm-ZP binding, whereas fragment Arg-32 to Thr-155 showed much weaker interaction with ZP4. Mutation of *N*-glycosylated Asn-146 to Asp in the *N*-terminal fragment Arg-32 to Glu-178 of ZP3 did not interrupt the interaction of this fragment with ZP4, but it did reduce the inhibitory activity of the complex for sperm-ZP binding. In contrast, mutation of *N*-glycosylated Asn-124 to Asp did not significantly reduce the activity. Taken together, these results suggest that one of the ZP4 binding sites exists in the flexible hinge region of ZP3 and that the *N*-glycosylation in this region is involved in the sperm binding.

## 1. Introduction

Mammalian oocytes are surrounded by the zona pellucida (ZP), a transparent envelope that mediates several critical aspects of fertilization, including species-selective sperm recognition and blocking of polyspermy [1,2,3,4,5]. Depending on the species, the ZP consists of either three or four kinds of glycoproteins. Bovine and porcine ZPs comprise three glycoproteins (ZP2, ZP3, and ZP4) [6]; mouse ZP also consists of three glycoproteins (ZP1, ZP2, and ZP3), whereas human, rat, hamster, rabbit, monkey, and cat ZPs consist of four (ZP1, ZP2, ZP3, and ZP4) [7,8]. All ZP proteins contain a domain called the ZP domain, which consists of ~260 amino acids and contains eight conserved Cys residues [9]. Among mammalian species, those possessing four ZP proteins are significantly more common than those possessing three.

In a previous study, we isolated bovine ZP from ovaries and partially separated an endo-β-galactosidase-digested bovine ZP into three components by reverse-phase high-performance liquid chromatography [6,10]. ZP4 exhibited the strongest inhibitory activity for sperm-ZP binding among the three partially purified components. In pigs, ZP4 contaminated with trace amounts of ZP3 showed sperm-binding activity, whereas purified ZP4 did not [11]. To test if this effect is also seen in bovines, we established a baculovirus-Sf9-based cell expression system of bovine ZP glycoproteins ZP3 and ZP4, both alone and in combination. Neither recombinant bovine ZP3 nor ZP4 exhibited any inhibitory activity for sperm-ZP binding; however, this activity was seen with the recombinant bovine ZP3/ZP4 complex co-expressed in Sf9 cells [12]. Taken together, these results suggest that ZP3/ZP4 heterocomplex formation is necessary for the sperm-binding activity of the ZP glycoproteins in both pigs and bovines.

In bovines, the major neutral *N*-linked chain of ZP consists of only one structure, a high-mannose-type chain containing five mannose residues [13]. α-Mannosyl residues at non-reducing termini are essential for the sperm-binding activity of bovine ZP [14]; the participation of *O*-linked chains in sperm binding has not yet been investigated. The involvement of sialic acids in sperm binding has also been reported [15]. Previously, we had reported that porcine recombinant ZP glycoproteins expressed in Sf9 cells have pauci- and high-mannose-type chains with α-mannosyl residues at non-reducing termini; these are capable of binding to bovine but not to porcine sperm [16]. Recombinant bovine ZP glycoproteins expressed in Sf9 cells also have sugar chains with α-mannosyl residues at non-reducing termini [12]. More recent analyses in an assay system using glycolipid analogues revealed that bovine sperm prefers α-mannosyl residues [17]. These results support a significant role for α-mannosyl residues in bovine sperm recognition of the ZP.

The ZP domain is responsible for the polymerization of proteins into filaments, as reported for mouse proteins ZP2 and ZP3 and the human Tamm-Horsfall protein [18]. Regardless of species, the ZP domain consists of three regions: the *N*-terminal subdomain (ZP-N), the *C*-terminal subdomain (ZP-C), and a flexible region connecting the two subdomains (hinge region). ZP precursors are synthesized as transmembrane proteins, with interactions between the internal hydrophobic patch (IHP) and external hydrophobic patch (EHP) inhibiting premature assembly of ZP proteins [19]. ZP precursors are processed at consensus furin cleavage sites between IHP and EHP, where mature ZP polypeptides containing IHP are separated from propeptides containing EHP and transmembrane domains, followed by initiation of ZP assembly. Protein crystal structures of a chicken homolog of mammalian ZP3 precursor revealed that IHP and EHP form anti-parallel β-sheets in the ZP precursors [20]. Thus, in mature ZP proteins, the loss of interaction between IHP and EHP triggers ZP protein assembly through an unknown mechanism.

**Figure 1:**
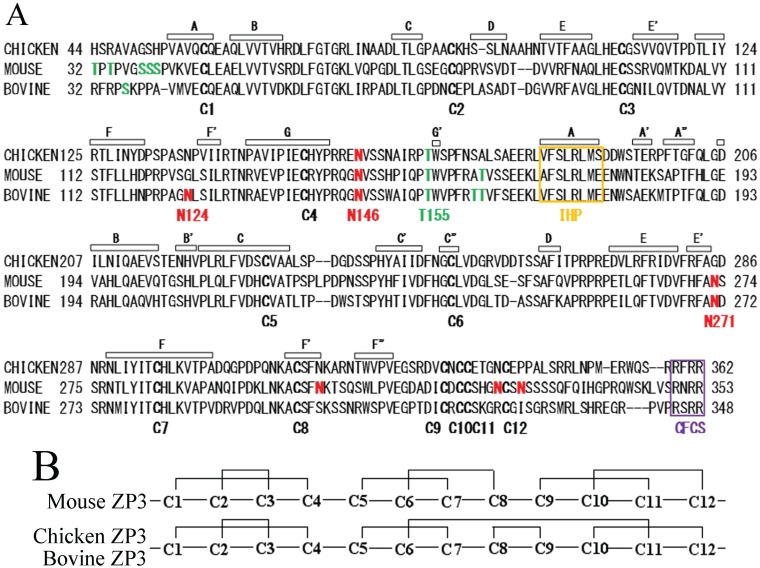

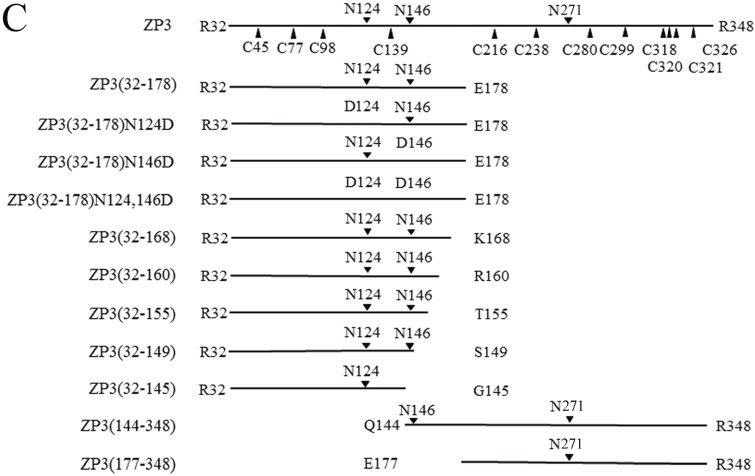
Primary structure of bovine zona pellucida glycoprotein-3 (ZP3) mature polypeptide. (**A**) Sequence alignment of bovine ZP3 mature polypeptide and the corresponding regions of chicken and mouse ZP3. Amino acid residues are numbered based on the translational initiation Met as 1. Cys residues (C1 to C12) are indicated in bold. The β-strands are indicated by open bars based on the structure of chicken ZP3 precursor [20]. Internal hydrophobic patch (IHP) and consensus furin cleavage sites (CFCS) are indicated by an orange box and a purple box, respectively. *N*-Glycosylated Asn residues are shown in red. *O*-Glycosylated Ser and Thr residues of chicken and mouse ZP3 and putative *O*-glycosylated Ser and Thr of bovine ZP3 are shown in green; (**B**) Disulfide bond patterns of mouse, chicken, and bovine ZP3; The 12 Cys residues C1 to C12 correspond to C1 to C12 shown in (**A**); respectively. Two types of disulfide bond pattern have been found in the *C*-terminal region containing C5 to C12. The putative disulfide bond pattern of bovine ZP3 is the same as that of chicken ZP3, although the disulfide pattern of bovine ZP3 has not been determined yet; (**C**) Schematic representation of the primary structure of bovine ZP3, along with all fragments examined in this study. The horizontal bars indicate polypeptides of recombinant proteins. *N*- and *C*-terminal amino acid residues of each recombinant protein are shown on the left and right sides of the bars, respectively; they are numbered based on the translational initiation Met as 1. Filled arrowheads show the positions of Cys (C) residues. Filled triangles show the positions of *N*-glycosylated Asn (N) residues. Asn (N) residues were mutated to Asp (D) in ZP3(32–178)N124D, ZP3(32–178)N146D, and ZP3(32–178)N124,146D fragments.

In mouse ZP3, ZP-N (Val-42 to Arg-143, see Figure 1A) has been expressed using bacterial expression systems and has been shown to self-assemble into filaments [21]. In chicken homologs of mammalian ZP1 and ZP3, ZP-C was revealed to interact with ZP3 and ZP1, respectively [22]. Taken together, these data suggest that ZP-C interacts with other ZP components, whereas ZP-N forms self-assembled filaments.

In bovines, ZP3 and ZP4 form sperm-binding active hetero-complexes. The region *N*-terminal to the ZP domain of ZP4 is dispensable for the formation of the sperm-binding active ZP3/ZP4 complex [12]. No studies on the interaction sites of bovine ZP3 and ZP4 have been published since this report. In this study, we focused on identification of ZP4-interaction sites on ZP3.

## 2. Results and Discussion

### 2.1. Identification of ZP4-Interaction Sites on ZP3

The disulfide bond pattern of bovine ZP3 has yet to be determined. However, sequence similarities between bovine ZP3 and porcine ZP3 suggest that the disulfide bond pattern of bovine ZP3 is the same as that of porcine ZP3 as well as that of chicken ZP3 (Figure 1B) [23]. The *N*-terminal region Val-41 to Arg-143 of bovine ZP3 containing four Cys residues at 45, 77, 98, and 139 (translational initiation Met is numbered as (1) corresponds to ZP-N. As the interaction between IHP and EHP has been shown to inhibit premature assembly of ZP proteins [19], IHP may be involved in the interaction of ZP3 with ZP4. The IHP is located in the region spanning Val-170 to Glu-177 (Figure 1A). The region from IHP to the *C*-terminus of bovine ZP3, containing eight Cys residues at 216, 238, 280, 299, 318, 320, 321, and 326 corresponds to ZP-C. Further fragmentation of ZP-N and ZP-C is not possible due to the disulfide bonds. The sequence similarities (57% identity) and the same disulfide bond pattern between bovine ZP3 and chicken ZP3 suggest that secondary and tertiary structures of bovine ZP3 are similar to those of chicken ZP3 (see Figure 1A for β-strands). Based on X-ray crystallographic models of the chicken ZP3 precursor, residues 143 to 169 of bovine ZP3 are thought to function as the flexible hinge region, although the three-dimensional structure of this region has yet to be determined [20]. Using these structural features as a starting point, fragments of the mature bovine ZP3 polypeptide spanning the *N*-terminus of mature ZP3 to IHP (Arg-32 to Glu-178), the *N*-terminus without the IHP (Arg-32 to Lys-168) and the *N*-terminus truncated prior to the hinge region (Arg-32 to Gly-145), along with *C*-terminal fragments Glu-177 to Arg-348 and Gln-144 to Arg-348 were expressed using a baculovirus-Sf9 cell system (Figure 1C). Each ZP3 fragment was tagged with six histidine residues (His-tag) followed by S-tag on the *N*-terminus of the protein and co-expressed with ZP4 containing an *N*-terminal FLAG-tag and an S-tag by co-infecting Sf9 cells with the corresponding recombinant baculoviruses. ZP3 fragments were pulled down using TALON affinity resin specific for the His-tag, whereas co-precipitated ZP4 was detected with an antibody specific to FLAG-tag.

**Figure 2:**
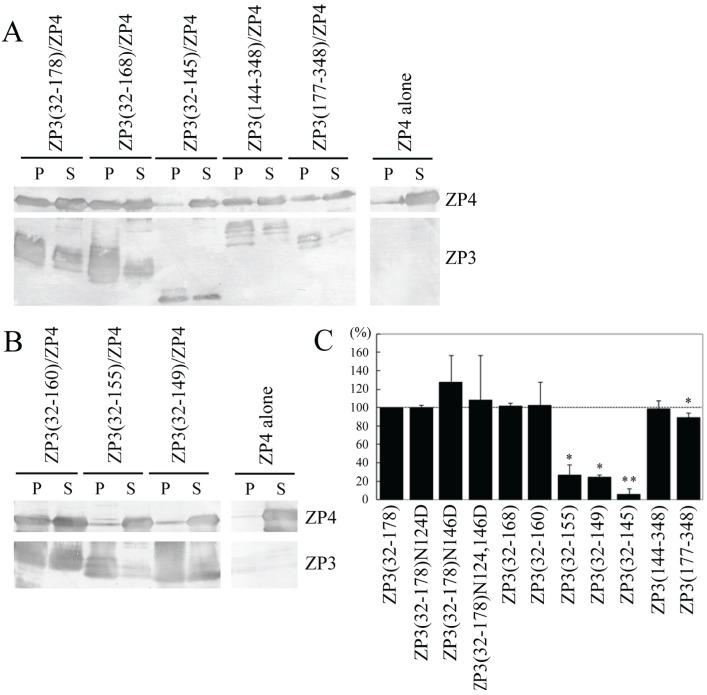
Co-precipitation of ZP4 with ZP3 fragments. (**A**,**B**) Detection of *N*-terminally FLAG- and S-tagged ZP4 co-precipitated with *N*-terminally His- and S-tagged ZP3 fragments by Western blot. ZP4 was expressed alone (ZP4 alone) or in combination with ZP3 fragments (indicated above each panel). The recombinant ZP3 fragments were pulled down using TALON resin. FLAG-tagged ZP4 in the pellet (P) and in the supernatant (S) was detected with an antibody specific to FLAG-tag (upper panels, ZP4); His-tagged ZP3 fragments in the P and the S were detected with an antibody specific to His-tag (lower panels, ZP3); (**C**) Percentage of ZP4 co-precipitated with each ZP3 fragment among the total ZP4 recovered in the pellet and supernatant. Percentages were quantified as described in the Experimental Section. The percentage of ZP4 co-precipitated with ZP3(32–178) is represented as 100%. Experiments were performed at least three times. Data are shown as mean ± S.D. Statistically significant differences, relative to 100% expression, are indicated as *p* > 0.05 (*) and *p* > 0.01 (**).

When ZP4 was expressed alone, only a small quantity of ZP4 was precipitated by TALON resin (Figure 2A). This quantity was significantly increased when co-expressed with ZP3(32–178), ZP3(32–168), ZP3(144–348) or ZP3(177–348); however, when ZP4 was co-expressed with ZP3(32–145), the quantity of precipitated ZP4 was similar to that of ZP4 expressed alone (Figure 2A). Relative quantities of precipitated ZP4 were calculated based on the quantities recovered when ZP4 was expressed alone and when it was co-expressed with ZP3(32–178), respectively, as determined using NIH Image J software. The percentages of co-precipitated ZP4 were not significantly different between ZP3(32–178) and ZP3(32–168), suggesting that the IHP of ZP3 is not essential for the interaction of ZP3 with ZP4. In contrast, the percentage of co-precipitated ZP4 was dramatically reduced to near basal levels when co-expressed with ZP3(32–145), suggesting that the region from 146 to 168 is necessary for the interaction of ZP3(32–178) with ZP4. The quantity of ZP4 recovered following co-expression with ZP3(177–348) was significantly lower than that of ZP4 co-expressed with ZP3(144–348); however, the difference in percentages was small, suggesting that one of the ZP4 binding sites is located in the region 177–348, making region 144–176 dispensable for the ZP4 binding of ZP3(144–348). Further efforts to identify the region within the 177–348 fragment responsible for ZP4 binding were not performed, as we instead chose to focus on the ZP4 interaction site in the region 146 to 168 of ZP3.

Evidence of a ZP4 binding site in the hinge region of ZP3 (residues 146–168) was further investigated by co-precipitation of ZP4 with each of the following ZP3 fragments: ZP3(32–160), ZP3(32–155), and ZP3(32–149) (Figure 1C). The co-precipitation of ZP4 with ZP3(32–160) was similar to that of ZP3(32–168) (Figure 2B). Further deletion from 156 to 160 dramatically reduced the co-precipitation of ZP4; additional deletions between residues 150 and 155 failed to restore the level of co-precipitated ZP4. These results suggest that the region spanning 161 to 178 is not necessary for the interaction of ZP3(32–178) with ZP4, whereas residues 156 to 160 are critically important for the binding of ZP4 to ZP3(32–178). Further deletion from 146 to 149 reduced the quantity of precipitated ZP4 by a small but significant extent, suggesting that the region from 146 to 149 is also involved in the ZP4 binding of ZP3.

Among residues 156–160, Arg-160 is the least conserved, with Ser, Trp, and His residues observed at this site in other mammals. Val-157 is almost completely conserved, although Leu is also found in some mammals. Trp-156, Pro-158, and Phe-159 are completely conserved across all mammals described to date; this strong conservation suggests that these residues are likely important for the binding of ZP4 to ZP3(32–178).

Our results indicating an interaction between the *C*-terminal half of ZP3 and ZP4 are consistent with previous reports describing avian homologs of ZP3. In addition to this region, these data suggest that there is also an additional ZP4-interaction site in the flexible hinge region of bovine ZP3.

### 2.2. Involvement of the Hinge Region of ZP3 in the Sperm-Binding Activity of the ZP3/ZP4 Complex

**Figure 3:**
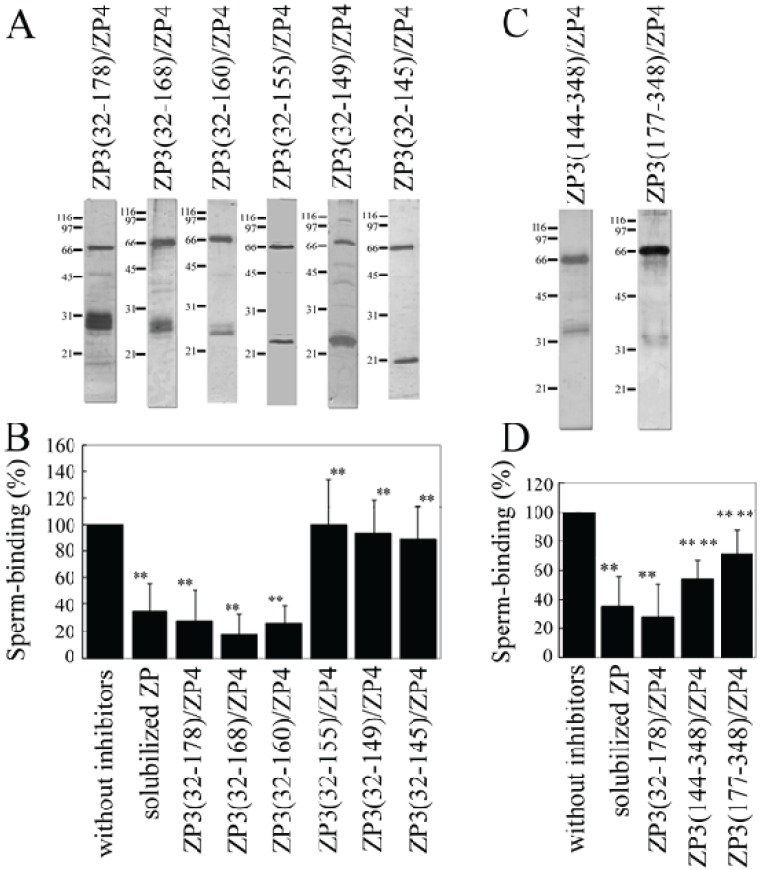
Inhibitory activity of a mixture of ZP3 fragment and ZP4 for the binding of bovine sperm to the zona pellucida (ZP). (**A**,**C**) SDS-polyacrylamide gel electrophoresis of a mixture of each ZP3 fragment and ZP4. *N*-terminally His-tagged ZP4 and an *N*-terminally His-tagged ZP3 fragment (indicated above each panel) were co-expressed in Sf9 cells, and the mixture was partially purified using TALON resin specific to His-tag. Gels were then silver-stained, with molecular mass standards (kDa) indicated on the left side of each panel. Bands at ~66 kDa correspond to ZP4, and the bands at 21 to 31 kDa in (**A**) and the bands at ~35 kDa in (**C**) correspond to ZP3 fragments; **(B**,**D**) Inhibitory activity of the mixture of ZP3 fragment and ZP4 for sperm-ZP binding. The number of sperm binding to ZP-coated wells in the absence of inhibitors was designated as 100%. Assays were repeated at least three times. Data are presented as means ± S.D. with statistical significance relative to “without inhibitors” and “ZP3(32–178)/ZP4” (asterisks on the left side and the right side of S.D. bars, respectively) indicated as *p* > 0.05 (*) and *p* > 0.01 (**).

A mixture of each of the ZP3 fragments and ZP4 was partially purified using TALON resin (Figure 3A,C) and used to test the inhibitory activity of each of the mixtures for the binding of bovine sperm to plastic wells coated with solubilized bovine ZP. The number of bovine sperm binding to ZP-coated wells was reduced to ~35% in the presence of solubilized bovine ZP. Neither the ZP3 fragment nor the ZP4 alone significantly inhibited sperm-ZP binding. In contrast, the ZP3(32–178)/ZP4 mixture significantly inhibited binding (Figure 3B). We next tried to identify a region on ZP3(32–178) involved in the formation of a sperm-binding active complex with ZP4. The co-expressed mixtures of ZP3(32–168)/ZP4 and ZP3(32–160)/ZP4 showed inhibitory activities for sperm-ZP binding that were similar to those of ZP3(32–178)/ZP4, whereas ZP3(32–155)/ZP4, ZP3(32–149)/ZP4, and ZP3(32–145)/ZP4 failed to significantly inhibit sperm-ZP binding. Thus, the inhibitory activity seen here is consistent with that of the co-precipitation result, confirming our previous result showing that the formation of the ZP3/ZP4 complex is necessary for its sperm-binding activity [12] and further suggesting that residues 161 to 178 are not necessary for the formation of the sperm-binding active ZP3/ZP4 complex.

The combinations of ZP3(144–348)/ZP4 and ZP3(177–348)/ZP4 significantly inhibited sperm-ZP binding, although the inhibitory activity of ZP3(177–348)/ZP4 was significantly lower than that of ZP3(144–348)/ZP4 (Figure 3D). These results suggest that the *C*-terminal region of ZP3 can form sperm-binding active complexes with ZP4 and that the region spanning residues 144 to 176 is involved in the formation of complexes possessing higher sperm-binding activity. Similarly, the inhibitory activity of ZP3(144–348)/ZP4 for sperm-ZP binding was significantly lower than that of the ZP3(32–178)/ZP4 mixture, suggesting that residues 144 to 176 contribute more to the sperm-binding activity of the ZP3(32–178)/ZP4 mixture than to the activity of ZP3(144–348)/ZP4.

### 2.3. Involvement of N-Linked Glycans on ZP3(32–178) in the Formation of Sperm-Binding Complexes

**Figure 4:**
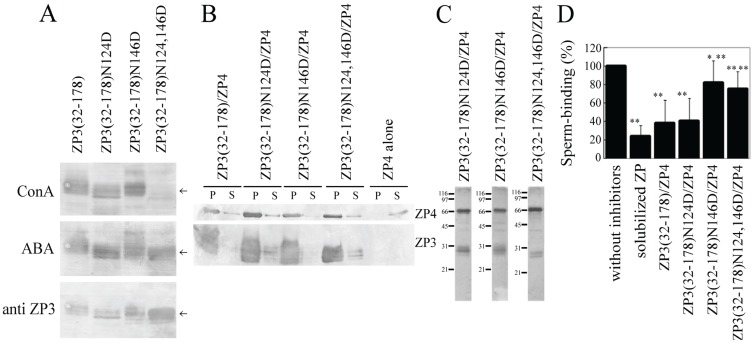
Effect of *N*-glycosylation site mutation of the ZP3 fragment on active sperm-binding complex formation between ZP3 fragment and ZP4. (**A**) Lectin blots of *N*-glycosylation site mutants of the ZP3(32–178) fragment. ZP3(32–178) fragment with Asn-124 mutated to Asp (ZP3(32–178)N124D), Asn-146 mutated to Asp (ZP3(32–178)N146D), and both Asn residues mutated to Asp (ZP3(32–178)N124,146D) as well as ZP3(32–178) were subjected to lectin blots. The same membrane was probed three times with Concanavalin A (ConA), *Agaricus bisporus* agglutinin (ABA), and, finally, anti-porcine ZP3 antiserum (anti ZP3), as described in the Experimental Section. ZP3 fragments are indicated by arrows; **(B**) Effect of *N*-glycosylation site mutation on ZP4 co-precipitation. *N*-terminally FLAG- and S-tagged ZP4 were expressed alone (ZP4 alone) or in combination with *N*-terminally His- and S-tagged ZP3 fragments (indicated above each panel). The recombinant ZP3 fragments were pulled down by TALON resin specific to His-tag. FLAG-tagged ZP4 in the pellets (P) and in supernatants (S) were detected with an antibody specific to FLAG-tag (upper panel, ZP4); His-tagged ZP3 fragments in the P and the S were detected with an antibody specific to His-tag (lower panel, ZP3). Percentages of co-precipitated ZP4 are shown in Figure 2C; (**C**) SDS-polyacrylamide gel electrophoresis of mixtures of *N*-glycosylation site mutants of ZP3(32–178) fragment and ZP4. Gels were then silver-stained, with molecular mass standards (kDa) indicated on the left side of each panel. Bands at ~66kDa correspond to ZP4; bands at 26 to 31kDa correspond to ZP3 fragments; (**D**) Inhibitory activity of the mixture of ZP3 fragment and ZP4 for sperm-ZP binding. The number of sperm binding to the ZP-coated well in the absence of inhibitors was designated as 100%. Assays were repeated at least three times. Data are presented as means ± S.D. with statistical significance relative to “without inhibitors” and “ZP3(32–178)/ZP4” (asterisks on the left side and the right side of S.D. bars, respectively) indicated as *p* > 0.05 (*) and *p* > 0.01 (**).

ZP3(32–178) has two *N*-glycosylation sites at Asn-124 and Asn-146. Here, we mutated each of these sites, either alone or in combination, to Asp. Concanavalin A recognized both ZP3(32–178) and ZP3(32–178) with Asn-124 mutated to Asp (ZP3(32–178)N124D), as well as ZP3(32–178) with Asn-146 mutated to Asp (ZP3(32–178)N146D) (Figure 4A), indicating that both Asn residues are *N*-glycosylated. Concanavalin A did not recognize ZP3(32–178) with both Asn-124 and Asn-146 mutated to Asp residues (ZP3(32–178)N124,146D) (Figure 4A), confirming the mutation of the two *N*-glycosylated Asn residues. Mutation of either Asn-124 or Asn-146 to Asp failed to reduce the quantity of ZP4 co-precipitated with ZP3(32–178) (Figure 4B and Figure 2C), indicating that *N*-glycosylation is not necessary for complex formation between ZP3(32–178) and ZP4. This result further indicates that these mutations did not destroy the steric structure of ZP3(32–178), since ZP3(32–178) retained the activity for the formation of a complex with ZP4 after the *N*-glycosylation sites were mutated.

Co-expressed mixtures of ZP3(32–178)N124D and ZP4 inhibited sperm-ZP binding at levels similar to ZP3(32–178)/ZP4, whereas the mixture of ZP3(32–178)N146D and ZP4 exhibited significantly lower levels of inhibitory activity than ZP3(32–178)/ZP4 (Figure 4C,D). Mutation of both Asn residues to Asp also significantly reduced the inhibitory activity of the ZP3(32–178)/ZP4 mixture (Figure 4D). Thus, the mutation of Asn-146 to Asp did not reduce the co-precipitation of ZP4, but it did reduce the sperm-binding activity of the complex, suggesting that *N*-glycan linked to Asn-146 is involved in the sperm-binding activity of ZP3(32–178)/ZP4.

When chicken ZP3 was analyzed by X-ray crystallography, the single *N*-glycosylation site corresponding to Asn-146 of bovine ZP3 was mutated to Gln; however, the recombinant chicken ZP3 without *N*-glycans still retained significant sperm-binding activity [20]. On the other hand, mutation of a single *O*-glycosylation site corresponding to Thr-155 in bovine ZP3 to Ala reduced the sperm-binding activity. As bovine ZP3 does not, by itself, inhibit sperm-ZP binding [12], the mechanisms regulating the binding of sperm to ZP might differ in bovines and chickens.

In combination with previous reports detailing the structure of chicken ZP3, the data presented herein suggest that glycans linked to the flexible hinge region are involved in sperm binding *in vitro*. Further studies will be necessary to determine whether the *N*-glycan at Asn-146 of bovine ZP3 binds to sperm directly or if the absence of an *N*-glycan at Asn-146 has an allosteric effect on the structure of the ZP3(32–178)/ZP4 complex.

## 3. Experimental Section

### 3.1. Construction of Recombinant Baculovirus Transfer Plasmids for Bovine ZP Proteins

Plasmid constructions for His- and S-tagged ZP4 as well as for FLAG- and S-tagged ZP4 have been previously reported [12]. The constructs encode Lys-25 to Arg-464 of ZP4 with the translation initiation Met numbered 1.

Preparation of cDNAs encoding *N*-terminal or *C*-terminal deletion mutants of ZP3 was performed by PCR using bovine ZP3 cDNA as a template. The 5' sense primers contained a *Bam*HI site, and the 3' antisense primers contained an *Xho*I site for ZP3(32–178) fragment, a *Hind*III site for the other *N*-terminal ZP3 fragments, or a *Bam*HI site for the *C*-terminal ZP3 fragments following a stop codon (Table 1). The PCR products were digested with the appropriate restriction enzymes and electrophoresed on 1% agarose gels; bands of expected sizes were excised from the gels, and the recovered DNA was ligated to a pBACgus6 vector (Novagen, Madison, WI, USA) digested with the same set of enzymes. The DNA sequences of the constructed plasmids were confirmed using a commercial DNA sequencing service (Macrogen, Fukuoka, Japan). The resulting recombinant ZP3 fragments contained *N*-terminal His- and S-tags.

Preparation of pBACgus6 plasmids encoding ZP3(32–178)N124D and ZP3(32–178)N146D was performed with the PrimeSTAR Mutagenesis Basal kit (TAKARA, Kyoto, Japan) using pBACgus6 encoding ZP3(32–178) as a template. All primers used in these experiments are listed in Table 1. pBACgus6 encoding ZP3(32–178)N124,146D was prepared by inserting a N146D mutation into ZP3(32–178)N124D using the same mutagenesis kit. The DNA sequences of the constructed plasmids were confirmed by sequencing.

**Table 1:** Primers used for amplification of ZP3 cDNA fragments and for site-directed mutagenesis.

	5' Sense 3'	5' Antisense 3'
ZP3(32–178)	CGGATCCTCGCTTCAGGCCATCAAAGCC	TCTCGAGCTACTCCTCCATCAGGCGCAG
ZP3(32–168)	CGGATCCTCGCTTCAGGCCATCAAAGCC	CAAGCTTACTTCTCCTCCGAGAACAC
ZP3(32–160)	CGGATCCTCGCTTCAGGCCATCAAAGCC	CAAGCTTACCTGAATGGCACCCAGGTG
ZP3(32–155)	CGGATCCTCGCTTCAGGCCATCAAAGCC	CAAGCTTAGGTGGGCTGGATGGCCCAG
ZP3(32–149)	CGGATCCTCGCTTCAGGCCATCAAAGCC	CAAGCTTAGCTACTCACATTGCCCTGCC
ZP3(32–145)	CGGATCCTCGCTTCAGGCCATCAAAGCC	CAAGCTTAGCCCTGCCTGGGGTAGTG
ZP3(144–348)	CGGATCCTCAGGGCAATGTGAGTAGCTG	CGGATCCTTACCTGCGACTTCGGGGAAC
ZP3(177–348)	CGGATCCTGAGGAGAACTGGAGCGCCG	CGGATCCTTACCTGCGACTTCGGGGAAC
N124D	TGCAGGAGACCTGTCCATCCTGAGGA	GACAGGTCTCCTGCAGGGCGGGGGTT
N146D	GCAGGGCGATGTGAGTAGCTGGGCCA	CTCACATCGCCCTGCCTGGGGTAGTG

Restriction sites (*Bam*HI, *Xho*I and *Hind*III) and point mutation sites from AAT/C (Asn) to GAT/C (Asp) are indicated by single and double underlines, respectively.

### 3.2. Recombinant Baculovirus

The procedure for preparing recombinant viruses for His- and S-tagged ZP4 and FLAG- and S-tagged ZP4 has been previously reported [12].

Plasmid DNA preparations containing individual cDNAs of ZP3 mutants were transfected along with BacMagic DNA (Novagen) into Sf9 cells according to the manufacturer’s protocol. Sf9 cells were routinely propagated in Sf-900II serum-free medium (Invitrogen, Carlsbad, CA, USA). Sf9 cells (1.8 × 10^6^) were attached to flasks, infected with recombinant virus, and cultured in 2.5 mL of Sf-900II serum-free medium for 48 h at 27 °C. The culture supernatant fraction was then recovered and mixed with 12 μL of a 50% suspension of TALON resin (TAKARA). The mixture was shaken gently at room temperature for 1 h to allow binding of the recombinant proteins to TALON through their His-tags. The affinity beads were washed three times with phosphate-buffered saline (PBS, pH 7.4), followed by centrifugation. The pellet containing the beads was then prepared for SDS-polyacrylamide gel electrophoresis (PAGE). Expression and secretion of each recombinant protein into the culture supernatant was examined by Western blot analysis using an antibody specific to His-tag (GE Healthcare, Little Chalfont, UK).

### 3.3. Detection of the Complex Formation of the ZP3 Fragment and ZP4

FLAG- and S-tagged ZP4 were expressed either alone or in combination with each of the His- and S-tagged ZP3 fragments by infection of Sf9 cells (2.5 mL at 1.0 × 10^6^ cells/mL) with a corresponding baculovirus or a mixture of corresponding baculoviruses and a successive culture for 48 h at 27 °C. The culture media were centrifuged at 800× *g* for 5 min, and each supernatant was mixed with 12 μL of a 50% suspension of TALON resin. The mixture was gently shaken for 1 h at room temperature. The TALON resins were recovered as pellets by centrifugation at 2000× *g* for 1 min, and S-tagged proteins in the supernatants were recovered as pellets using S-protein agarose (Novagen). Pellets were then washed three times with PBS; each washing was followed by centrifugation at 2000× *g* for 1 min. Final pellets were prepared for SDS-PAGE. SDS-PAGE was performed under reducing conditions according to the Laemmli protocol [24]. Proteins were separated on 12.5% gels and transferred to Immobilon-P membranes (Millipore, Bedford, MA, USA) according to the method of Towbin [25]. The membranes were blocked with 3% bovine serum albumin (BSA) in Tris-buffered saline (TBS; 20 mM Tris-HCl, pH 7.5, 500 mM NaCl) for 1 h. FLAG-tagged ZP4 and His-tagged ZP3 fragments were detected with anti-FLAG M2 antibody (diluted to 4.8 μg/mL; Sigma, St. Louis, MO, USA) and anti-His antibody (diluted 1/3000; GE Healthcare), respectively, as primary antibodies and horseradish peroxidase-conjugated anti-mouse IgG antibody (diluted 1/2000; Sigma, St. Louis, MO, USA) as a secondary antibody. The blots were developed using 3,3',5,5'-tetramethyl benzidine (TMB) solution (KPL, Gaithersburg, MD, USA).

The membranes were scanned. Band intensities of ZP4 were quantified using Image J software. When ZP4 was expressed alone, a small quantity of ZP4 was precipitated. The quantity of this non-specifically precipitated ZP4 was subtracted from those of ZP4 co-precipitated with ZP3 fragments. The percentages of precipitated ZP4 were calculated as follows: (band intensity of ZP4 in the pellet)/[(band intensity of ZP4 in the pellet) + (band intensity of ZP4 in the supernatant)] × 100. The percentages of ZP4 co-precipitated with ZP3(32–178) varied according to experiments; however, those were designated as 100% as a standard.

### 3.4. Purification of Recombinant Proteins from Culture Supernatants

For large-scale protein production, Sf9 cells (200 mL at 1.0 × 10^6^ cells/mL) were infected with a corresponding recombinant virus or a mixture of corresponding recombinant viruses. After 48 h of culture in suspension, the medium was centrifuged at 800× *g* for 10 min, and the supernatant fraction was filtered through a 0.45 μm filter. The filtrate was sonicated and then stored at 4 °C.

For purification of His-tagged proteins, the filtered and sonicated supernatants were subjected to metal-chelation column chromatography using TALON resin equilibrated with column buffer (20 mM Tris-HCl, pH 7.9, 0.15 M NaCl) at a flow rate of 0.5 mL/min at 4 °C. The column was washed with 10-column volumes of the column buffer, and the bound protein was eluted with six-column volumes of column buffer containing 150 mM imidazole.

Protein concentrations were determined using Protein Assay Dye Reagent Concentrate (Bio-Rad, Hercules, CA, USA) with BSA as a standard. Protein yield was typically 15 µg from 200-mL culture.

### 3.5. Lectin Blot

Proteins were separated on 12.5% gels and transferred to Immobilon-P membranes. The membranes were blocked with TBS containing 3% BSA for 1 h and then incubated for 2 h with 1 μg/mL of biotin-conjugated Concanavalin A (Honen, Tokyo, Japan) in TBS containing 0.05% Tween 20 and 1 mM each of MgCl_2_ and CaCl_2_ (T-TBS). Membranes were washed three times for 15 min each with T-TBS and incubated for 1 h with 0.5 μg/mL of horseradish peroxidase-conjugated streptavidin (Sigma) in T-TBS. Membranes were then washed three times, followed by colour development using TMB. The membranes were stripped by incubating at 50 °C for 30 min in 62.5 mM Tris-HCl (pH 6.7) containing 2% SDS and 100 mM 2-mercaptoethanol and then used for the next lectin blot with *Agaricus bisporus* agglutinin (ABA, EY Laboratories, San Mateo, CA, USA) and, finally, with anti-porcine ZP3 antiserum [6].

### 3.6. Competitive Inhibition Assays

Competitive inhibition assays were performed in 96-well plates, as described previously [12]. Briefly, solubilized bovine ZP (0.2 μg in 50 μL of PBS) was adsorbed to each well of a 96-well plate (Nalge Nunc, Rochester, NY, USA). The wells were blocked with BSA. Frozen bovine sperm were thawed, washed twice, and then capacitated in pre-warmed (38.5 °C) Brackett and Oliphant (BO) solution without BSA [12,26]. The bovine sperm were then capacitated by incubation in BO solution containing BSA for 30 min. Capacitation and subsequent incubations were carried out at 38.5 °C under 2% CO_2_. Aliquots (50 μL) containing 4 × 10^5^ capacitated sperm were mixed with 50 μL of BO solution containing solubilized bovine ZP or each recombinant ZP protein and then incubated for 30 min in the presence of 0.3 µg inhibitor. The wells were rinsed three times with PBS, the pre-incubated sperm solutions were transferred into the wells, and the plates were incubated for 2 h. The wells were then washed three times with BO solution, 50 μL of 70% glycerol in PBS was added to each well; sperm bound to the wells were recovered by 20 strokes of vigorous pipetting. The number of sperm in 0.1 μL of suspension was determined using a haemocytometer. The number of sperm bound to the wells not coated with solubilized ZP (0 to 5) was subtracted from the number of sperm bound to the wells coated with solubilized ZP. The number of bound sperm in the control experiments without inhibitors varied from 90 to 200, with an average of 110.

### 3.7. Statistical Analysis

Welch’s *t*-test was used to determine whether the inhibitory activities of recombinant ZP proteins differed significantly. Differences were considered to be significant at *p* < 0.05.

## 4. Conclusions

Both the *N*- and *C*-terminal halves of bovine ZP3 can form sperm-binding active complexes with ZP4. The hinge region is necessary for the interaction of the *N*-terminal half of ZP3 with ZP4. *N*-linked glycans bound to the hinge region are involved in the sperm-binding activity of complexes consisting of the *N*-terminal half of ZP3 and ZP4, although the mechanism of this involvement, including whether it is direct or allosteric, remains poorly understood.
